# Developing and Preliminary Validating an Automatic Cell Classification System for Bone Marrow Smears: a Pilot Study

**DOI:** 10.1007/s10916-020-01654-y

**Published:** 2020-09-07

**Authors:** Hong Jin, Xinyan Fu, Xinyi Cao, Mingxia Sun, Xiaofen Wang, Yuhong Zhong, Suwen Yang, Chao Qi, Bo Peng, Xin He, Fei He, Yongfang Jiang, Haiyan Gao, Shun Li, Zhen Huang, Qiang Li, Fengqi Fang, Jun Zhang

**Affiliations:** 1 Clinical Laboratory, Sir Run Run Shaw Hospital, School of Medicine, Zhejiang University, Hangzhou, 310016 Zhejiang China; 2 Division of Medical Technology Development, Hangzhou Zhiwei Information & Technology Ltd., Hangzhou, 311121 China; 3 Department of Hematology, the Second Affiliated Hospital of Harbin Medical University, Harbin, 150086 China; 4 Department of Research and Development, Hangzhou Zhiwei Information & Technology Ltd., Hangzhou, 311121 China; 5 Department of Oncology, the First Affiliated Hospital of Dalian Medical University, Dalian, 116011 China

**Keywords:** Bone marrow smear, Differential cell count, Cell classification, Digital image

## Abstract

Bone marrow smear examination is an indispensable diagnostic tool in the evaluation of hematological diseases, but the process of manual differential count is labor extensive. In this study, we developed an automatic system with integrated scanning hardware and machine learning-based software to perform differential cell count on bone marrow smears to assist diagnosis. The initial development of the artificial neural network was based on 3000 marrow smear samples retrospectively archived from Sir Run Run Shaw Hospital affiliated to Zhejiang University School of Medicine between June 2016 and December 2018. The preliminary field validating test of the system was based on 124 marrow smears newly collected from the Second Affiliated Hospital of Harbin Medical University between April 2019 and November 2019. The study was performed in parallel of machine automatic recognition with conventional manual differential count by pathologists using the microscope. We selected representative 600,000 marrow cell images as training set of the algorithm, followed by random captured 30,867 cell images for validation. In validation, the overall accuracy of automatic cell classification was 90.1% (95% CI, 89.8–90.5%). In a preliminary field validating test, the reliability coefficient (ICC) of cell series proportion between the two analysis methods were high (ICC ≥ 0.883, *P* < 0.0001) and the results by the two analysis methods were consistent for granulocytes and erythrocytes. The system was effective in cell classification and differential cell count on marrow smears. It provides a useful digital tool in the screening and evaluation of various hematological disorders.

**Electronic supplementary material:**

The online version of this article (10.1007/s10916-020-01654-y) contains supplementary material, which is available to authorized users.

## Introduction

The incidence of hematopoietic and lymphoid malignancies is increasing worldwide. [1] Bone marrow (BM) aspirate examination is a critical step in the initial work-up for hematological diseases. Differential counts of BM cells are requisite for diagnosis since the World Health Organization’s (WHO) classification of hematologic neoplasms relies on percentages of specific cell types. [1, 2] However, the process of manual differential count is labor-extensive, time-consuming, and often lacks consistency due to intra-observer variability. Therefore, there is an utmost need to develop a reliable method to assist conventional manual examination. [3, 4]

For the microscopic examination of peripheral blood smear, several instruments have been developed and proved to be efficient in digital morphological analysis, such as DM9600, DI-60, Cobas M511 and Vision Hema. [5–8] These systems have made promising advancements in automation, digitization, standardization and intellectualization of peripheral blood smear analysis. However, limited progress has been made in automation of BM smears due to the complexity of marrow specimens. [9–13] In addition, more technical challenges are present in the marrow specimen including the use of oil-immersion lens for slide digitization, scanning field selection, and development of proper focusing algorithms. [14]

The emerging technology of whole slide imaging (WSI) [15, 16] and artificial intelligence (AI) [17–19] applications are revolutionized in improving the efficiency of cytopathological examinations. [20, 21] There are systems currently under investigation for analyzing BM smears, such as Vision Bone Marrow [22] and Scorpio Full Field BMA. [23] In this study, we take advantages of a high-resolution BM smear scanning device and advanced deep learning algorithms for cell classification. The efficiency and reliability of the system are validated, and initial application in clinical hematology laboratories are analyzed.

## Methods

### Study design and sample collection

This study was simultaneously completed in two clinical hematology laboratories in China. First, we retrospectively collected more than 3000 BM aspirate smears at Sir Run Run Shaw Hospital (SRRSH) affiliated to Zhejiang University School of Medicine from June 2016 to December 2018 to improve the hardware design of the system and train the AI algorithms. Cell classification utilizing AI algorithms embedded in the system was preliminarily validated with 145 BM smears collected from SRRSH. The system’s analysis ability for BM smears was tested by assessing differential count results during the clinical usage using 124 smears retrospectively collected from the Second Affiliated Hospital of Harbin Medical University between April 2019 and November 2019. All the aspirated smears were well stained by Wright-Giemsa protocol. The quality of the smears met the requirements of the National Guide to Clinical Laboratory Procedures (NGCLP, fourth edition) [24] or the International Council for Standardization in Hematology (ICSH) recommendation. [25] Smears completely diluted by peripheral blood, with unclear patient information, or considered to be inadequate by investigators were excluded.

### Design of the system

#### Hardware

**Fig. 1:**
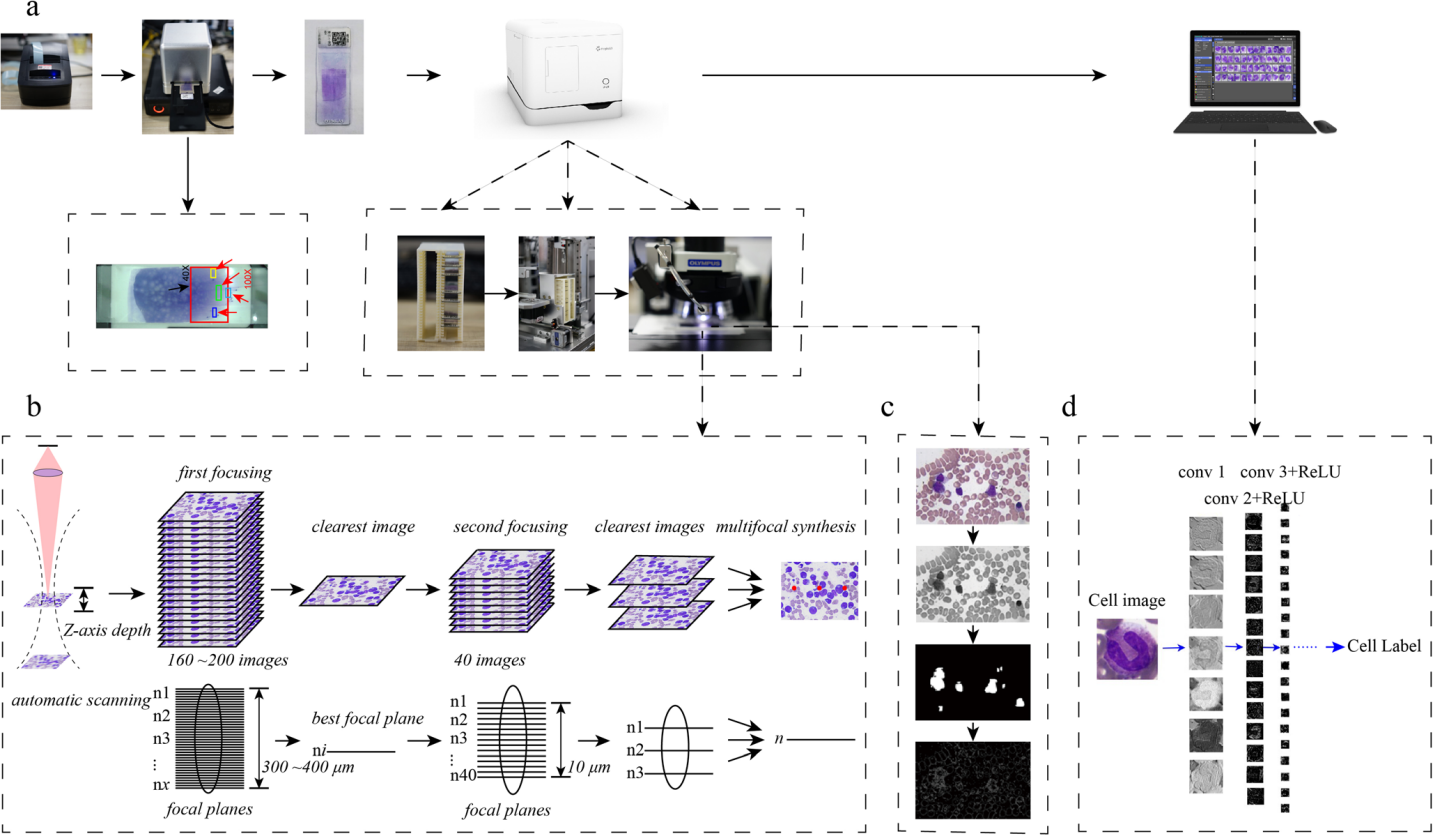
**Hardware and working principle of the system.** a. Composition of the system, including a label printer, a global view box, a high-resolution digital image scanner and the pre-installed image managing and cell classification software in the computer. b. The autofocusing algorithm for obtaining clear images. c. Definition evaluation by the autofocusing algorithm. First, it finds the region of WBCs, then calculatesthe definition. Then, it finds the focusing position roughly by the mean square differenceand then uses the Canny operator for fine focusing. d. Artificial neural network for cell recognition. The network consisted of 27 layers and it automatically identified and labeled nucleated cells on the digitized BM smears by extracting cell features or eigenvalues within the network

To generate clear digital images of BM smears, a novel piece of automated scanning hardware was developed and named “*Morphogo*”. It consists of a label printer, a global view box for selecting analysis area, a slide holder that could accommodate 27 slides at a time, a scanner, and a computer (see Fig. 1a). The scanner consists of a mechanical control unit for loading and transferring slides, a microscopy unit installed with a 40 × objective (Plan N 40 × /0.65 FN22, resolution 0.42 μm, Olympus, Japan) and a 100 × objective (Plan N 100 × / 1.25 FN22, resolution 0.22 μm, Olympus, Japan), an oil-dropping unit, a light source unit and a camera with 4000 × 3000 pixels (E3ISPM12000KPA with 12MP 1/1.7“(7.40 × 5.55) SONY Exmor CMOC Sensor, ToupCam, China). Its structure was shown in the animation (**Online Resource** **1**). The computer configurations were as follows: CPU 3.7GHz (Core i9 10900X, Intel, USA), motherboard X299 SAGE (ASUS, China), memory 16G×4 (DDR4 2666MHz, ADATA, China), hard disk 1T (SN700 NVME M.2 2.5” 5400 rpm, WD, USA), graphics card chips 11GB × 3 (GeForce RTX 2080 Ti, NVIDIA, USA), LED monitor 1080p (C27F591FDC 27-inch. 1920 × 1080 pixels, Samsung, South Korea).

#### Algorithms

BM smears were manually prepared. To achieve the best clarity of obtained cell images, we designed an autofocusing algorithm to fit the variable thickness of cell layers on the slides. During autofocusing, the region of white blood cells (WBCs) was firstly found to avoid focusing on the impurities, and then definition was calculated. Secondly, a coarse focus point was determined by mean square deviation. Then, local pixel difference at the edge of located nucleated cells were measured by Canny operator which removed the noise background in cell images. Eventually acquisition was completed by determining the best focus with fine focusing. (see Fig. 1b, c).

To enable automatic detection and recognition of BM cells, we developed a series of algorithms for cell classification. These algorithms consist of three parts: cell localization, cell segmentation and cell recognition (classifier). a. Cell localization: From a microscopic point of view, nucleated cells on an aspiration smear were unevenly distributed and located at various heights, so it was difficult to accurately detect and locate the cells on the images. We transformed the original images into grayscale images and used the clustering algorithm to analyze and extract nucleated cells. b. Cell segmentation: According to the distribution of color range of nucleated cells and the fact that the color of red blood cells (RBCs) and the background are darker than nucleated cells in most scenarios, we utilized the k-mean and decision tree to achieve the accurate segmentation of nucleated cells on the images. c. Cell recognition: A 27-layered artificial neural network was constructed to automatically extract cellular features and to be used for cell classification after training (see Fig. 1d). The algorithm development used the following tools: Tensorflow 1.8.0, Scipy v1.0.0.and OpenCV 3.1.0 library.

#### Software

The software of the system includes three types of clients: acquisition terminal, review terminal and telepathology terminal.

The acquisition terminal was designed for users to acquire and process digital image of BM smear, to count cells and to generate reports. It consisted of the mechanical control module for initializing instrument’s self-inspection, slide loading and transferring, objective lens switching, and automatic oil dropping; the information and reporting module for setting up user accounts, filling slide information, selecting scanning area (both 40× and 100×), providing statistical results of cell count, issuing reports, communicating with other users; the image acquisition and processing module for 40× digital WSI acquisition and assembly, 100× image acquisition, detection of megakaryocytes on 40× WSI, detection of nucleated cells, segmentation and classification of nucleated cells on 100× images; and the image management module for display, modification and management of cell images.

The review terminal was designed for users to review results of slide acquisition and cell classification uploaded from the acquisition terminal. It enabled users to set up accounts, modify slide information, manage digital images, review differential count results, and issuing reports. Report templates and default diagnostic opinions were also customized.

The telepathology terminal was designed for remote diagnosis. It could be used for long-distance transmission of digital images of BM smears and it allowed communication between multiple users.

**Fig. 2:**
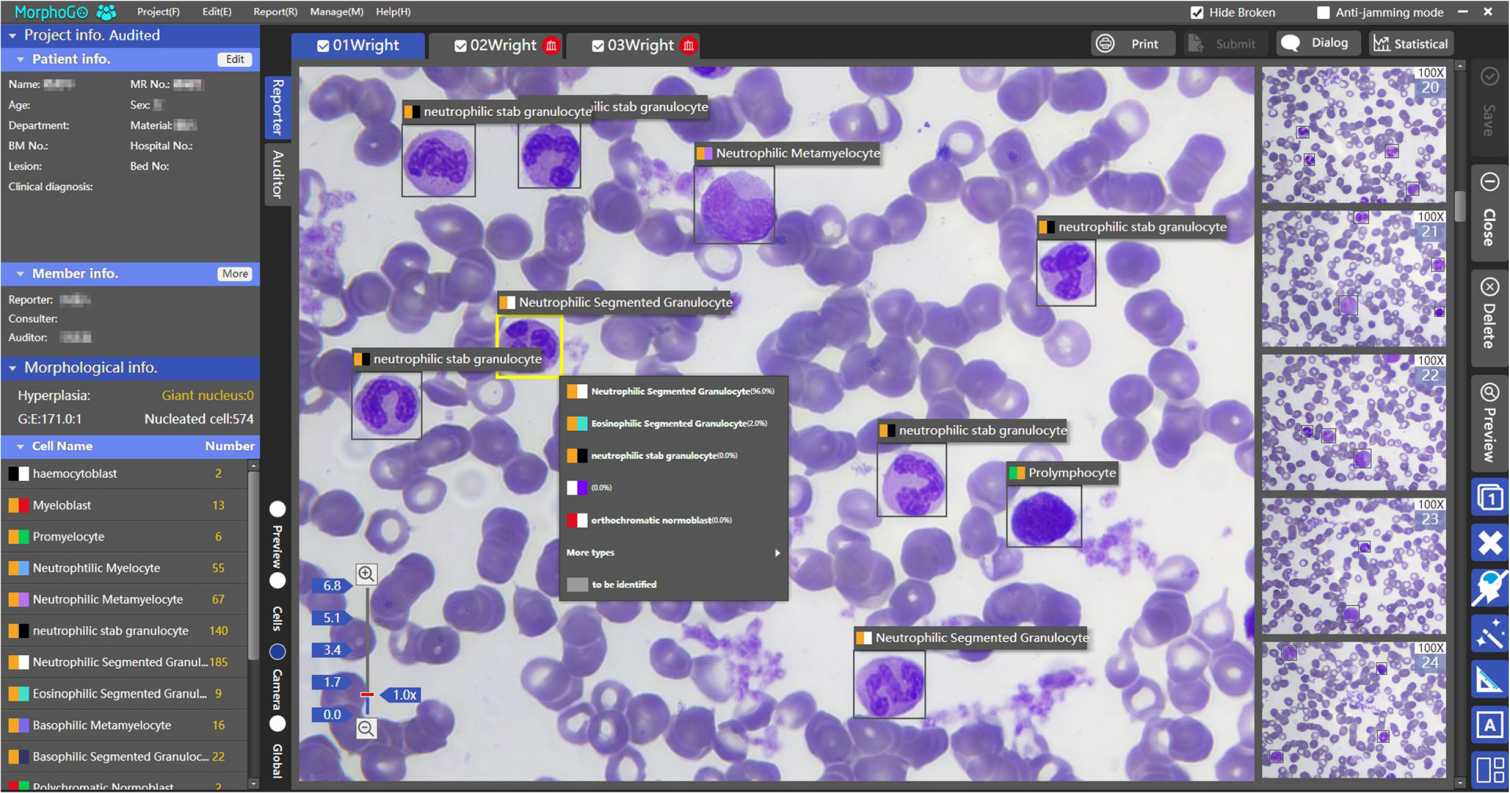
**Digital images (100**×**) and cell classification displayed in the software.** Digital images of a BM smear (574 cells counted). The system performed cell classification with AI algorithms and provided a list with the five most likely choices of cell types

**Fig. 3:**
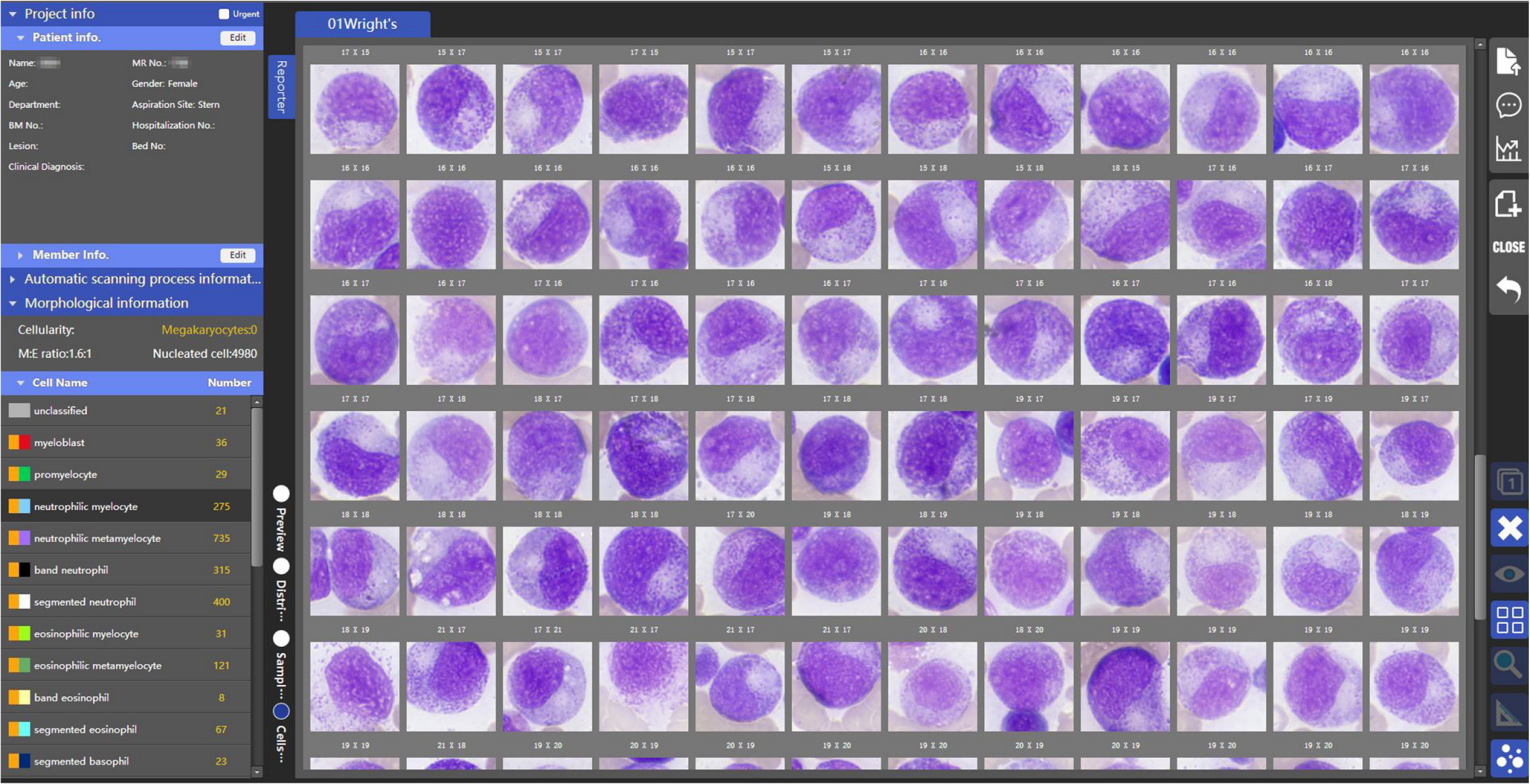
**Individual cell images gallery (100**×**) displayed in the software.** Granulocytic myeloid cells in a spectrum of maturation were classified by the algorithms in a digitized BM smear with confirmed diagnosis of multiple myeloma (4980 cells counted)

Both acquisition and review terminals allow users to display and modify digital slides and individual cell images (see Figs. 2, 3). When a user reviews the differential count results and the overall condition of the slide, the software provides selective communication functions like the annotation tool, the scale, the magnifier as well as a classification list with the five most likely choices of cell types recognized by the algorithms. (see Fig. 2).

#### Workflow

**Fig. 4:**
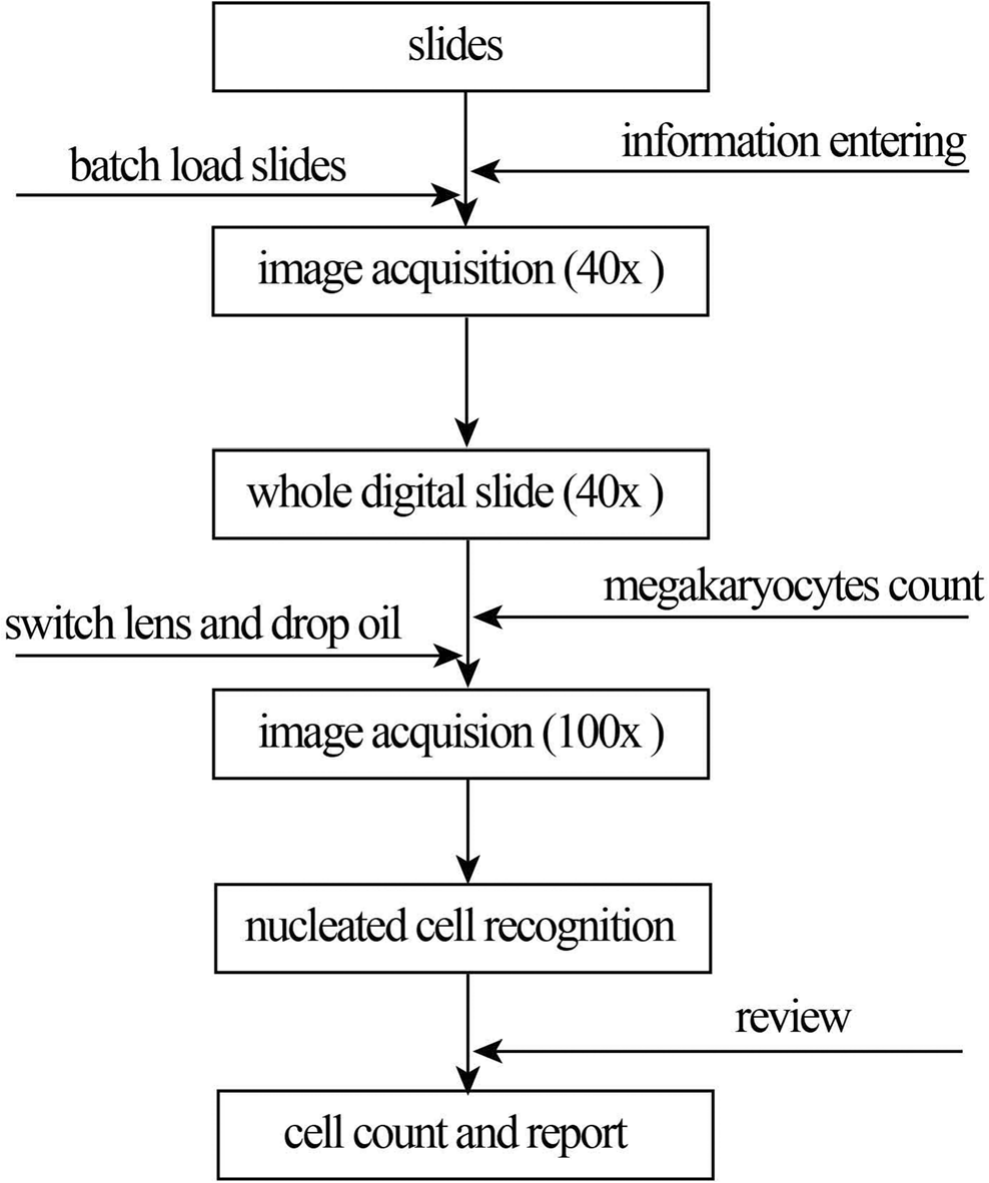
Digital workflow the system

The system digitizes BM smear, performs differential count and generates cytology reports. Workflow of the system is shown in Fig. 4. Detailed descriptions are shown in **supplemental material (Online Resource** **2****)**.

#### Work time

The time used for digitizing a slide was directly proportional to the analysis area selected for scanning and the number of nucleated cells to be acquired. The hardware supported WSI (44 mm × 22 mm) with 40× objective and cell acquisition with 100× objective. The scanning speed was 50 mm^2^/min with 40× objective (WSI, pixel size 0.17 ± 0.02 μm) and 20 images/min with 100× objective (image size 4000 × 3000 Pixels, pixel size 0.018 ± 0.005 μm). In general, it took about 32 min to complete generation of a WSI and acquisition of 500 cells from a slide (with both 40× and 100× objectives, see Fig. 2), and it took 105 min to count 5000 cells for a slide. (with both 40× and 100× objectives, see Fig. 3).

### Digitization of BM smears and acquisition of cell images

All the collected smears were digitized with the system in the clinical hematology laboratories of local hospitals and all samples were collected anonymously. Nucleated cells (sized at 500–600 × 500–600 pixels) in the acquired images were automatically localized, segmented and classified by the system, and then the AI-based classification results were reviewed by pathologists. 200–500 nucleated cells were captured and counted in each digitized smear. Nucleated cells acquired from smears were classified into 12 categories according to WHO’s classifications, [1] including myeloblasts, promyelocytes, myelocytes, metamyelocytes, neutrophils, eosinophils, basophils, monocytes, erythroblasts, lymphocytes, plasma cells, and other cells (broken cells or smudge cells, rare hematopoietic cells such as histiocytes, and non-hematopoietic cells).

### Training of the algorithm

We captured and labeled more than 600,000 cell images from SRRSH which were randomly assigned to two data set according to a ratio of 0.8:0.2 for training and verification of the algorithm. The training of algorithm was run on a server equipped with Intel Core i9 10,900X, 16G × 4 ADATA DDR4, NVIDIA GeForce RTX 2080 Ti cards, and CUDA Version 10.2. The algorithm was trained by multiple training batches, with a base training period of 50.8 h, and 25.8 h each time for additional cells. After repeated iterative training, an optimal algorithm for cell classification was obtained and internally verified. Moreover, the algorithm’s ability of cell classification was continuously being optimized while it was used in the clinical laboratories.

### Validation of cell classification ability of the system

Based on initial validation of the algorithm, we optimized and further validated the cell classification ability of the algorithm using another 145 smears collected from SRRSH. A total of 30,867 cell images were captured, classified by the system, and independently reviewed by pathologists with a consent as final results. The cell classification ability of the system was assessed by accuracy, sensitivity and specificity.

### Testing smear analysis ability of the system

To assess the system’s differential cell count ability for BM smears, we tested the system with 124 smears retrospectively collected from the Second Affiliated Hospital of Harbin Medical University. Detailed information of smears is described in the **supplemental material (Online Resource** **3****)**. The smears were automatically digitized and analyzed by the system. The results were reviewed and reported by the pathologists. When reporting results, nucleated cells acquired from each smear were classified into five series with proportions, including granulocytes, erythroid, lymphoid, monocytes, plasma cells. At the same time, the smears were examined by the pathologists using the microscope to produce manual differential count reports. Thus, for every smear, we obtained two groups of cell series proportions: cell series proportions analyzed by the system then reviewed by pathologists, and proportions resulted from pathologists’ manual differential count using the microscope.

The consistency between the two reporting methods were assessed by intraclass correlation coefficient (ICC), Passing-Bablok regression analysis [26, 27] and Bland-Altman plot [28].

### Statistical analysis

Numerical variables were described as mean and standard deviation (^−^*x* ± *s*). Cell classification data analysis was performed with Microsoft Excel version 2016 (Microsoft Corporation, Redmond, WA, USA) and Python 3.6.5 (Python Software Foundation) with a library of Pycm 2.1. [29] ICC for consistency evaluation of measurement methods were performed by SPSS v18.0 (IBM SPSS Statistics, IBM, Chicago, USA). Passing-Bablok regression analysis and Bland-Altman plot analysis, which established for agreement evaluation, were performed in NCSS v12.0 (NCSS, LLC, Kaysville, Utah, USA). All the tests were performed by two-tailed test, and *P* ≤ 0.05 was considered statistically significant.

## Results

For validation of cell classification, the overall accuracy was 90.1% (95% CI, 89.8–90.5%). Other indicators of the evaluation are shown in Table 1. These results demonstrated that the algorithms performed well in cell classification for BM smears at SRRSH. However, the sensitivities of cell classification varied for different stages of BM cells (Table 1). Some of the cell type such as promyelocytes show low sensitivity due to the overlap with myelocytes.

**Table 1:** Automatic cell classification ability of the system vs results reviewed by pathologists

**Overall Statistics**	**Accuracy (%)**	**95% CI (%)**	
	90.1	(89.8–90.5)	
Class:	Accuracy (%)	Sensitivity (%)	Specificity (%)
Myeloblasts	99.1	66.9	99.6
Promyelocytes	99.0	42.7	99.8
Myelocytes	97.5	78.3	98.9
Metamyelocytes	96.1	76.1	98.1
Neutrophils	97.6	97.2	97.8
Eosinophils	99.6	76.6	99.9
Basophils	99.8	72.8	99.9
Monocytes	98.0	95.2	98.8
Erythroblasts	97.9	73.2	98.7
Lymphocytes	97.0	95.0	97.5
Plasma Cells	99.2	88.5	99.3
Tissue and other cells	99.7	35.6	100.0

For testing of smear analysis ability, the reliability coefficient (ICC) between two different analysis methods were high for granulocytes, erythrocytes, lymphocytes (ICC ≥ 0.763, *P* < 0.0001), and slightly lower for monocytes and plasma cells (ICC ≤ 0.401, *P* < 0.0001, Table 2).

**Table 2:** ICC of two different analysis methods in smears

**Classes**	**ICC**	**95% CI**	***F*** **value**	***P*** **value**
Granulocytes	0.893	(0.851–0.924)	17.748	<0.0001
Erythrocytes	0.883	(0.837–0.916)	16.063	<0.0001
Lymphocytes	0.763	(0.678–0.827)	7.422	<0.0001
Monocytes	0.449	(0.297–0.579)	2.629	<0.0001
Plasma cells	0.368	(0.203–0.513)	2.165	<0.0001

Abbreviation: ICC, intraclass correlation coefficient

**Fig. 5:**
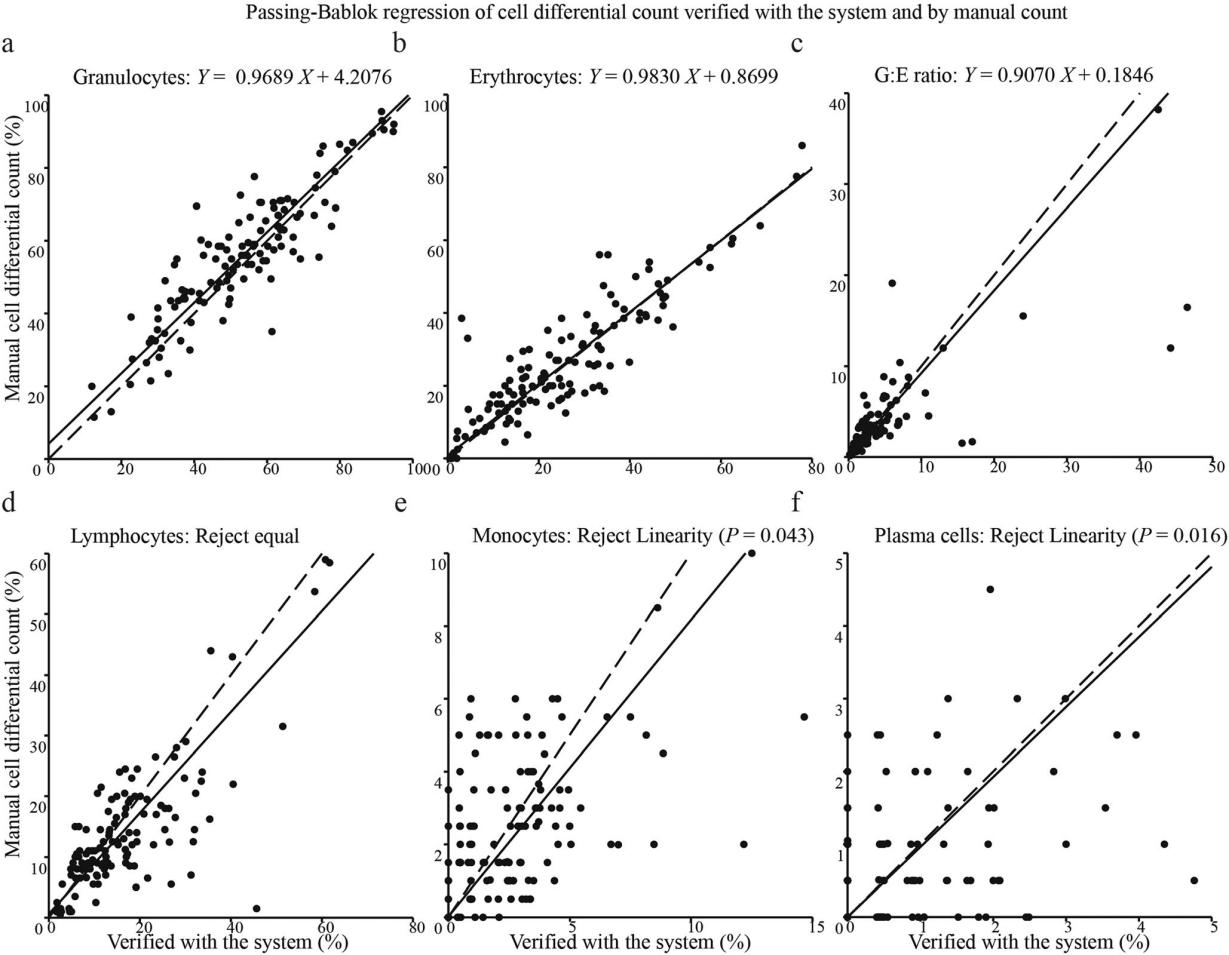
**Passing-Bablok regression analysis of cell series proportions reviewed by the pathologists with the system and with manual different count.** a–f: Passing-Bablok regression scatter plots and linear equations of granulocytes, erythrocytes, granulocytes: erythrocytes (G:E) ratio, lymphocytes, monocytes, plasma cells

Passing-Bablok regression analysis showed that there were consistencies of cell series proportions for granulocytes and erythrocytes, the regressions were: granulocytes *Y* = 0.9689 *X* + 4.2076, erythrocytes *Y* = 0.9830 *X* + 0.8699 (Fig. 5a, b). There was no consistency for lymphocytes (Reject equal, Fig. 5d), and there was no linearity for monocytes (*P* = 0.043, Fig. 5e) and plasma cells (*P* = 0.016, Fig. 5f).

**Fig. 6:**
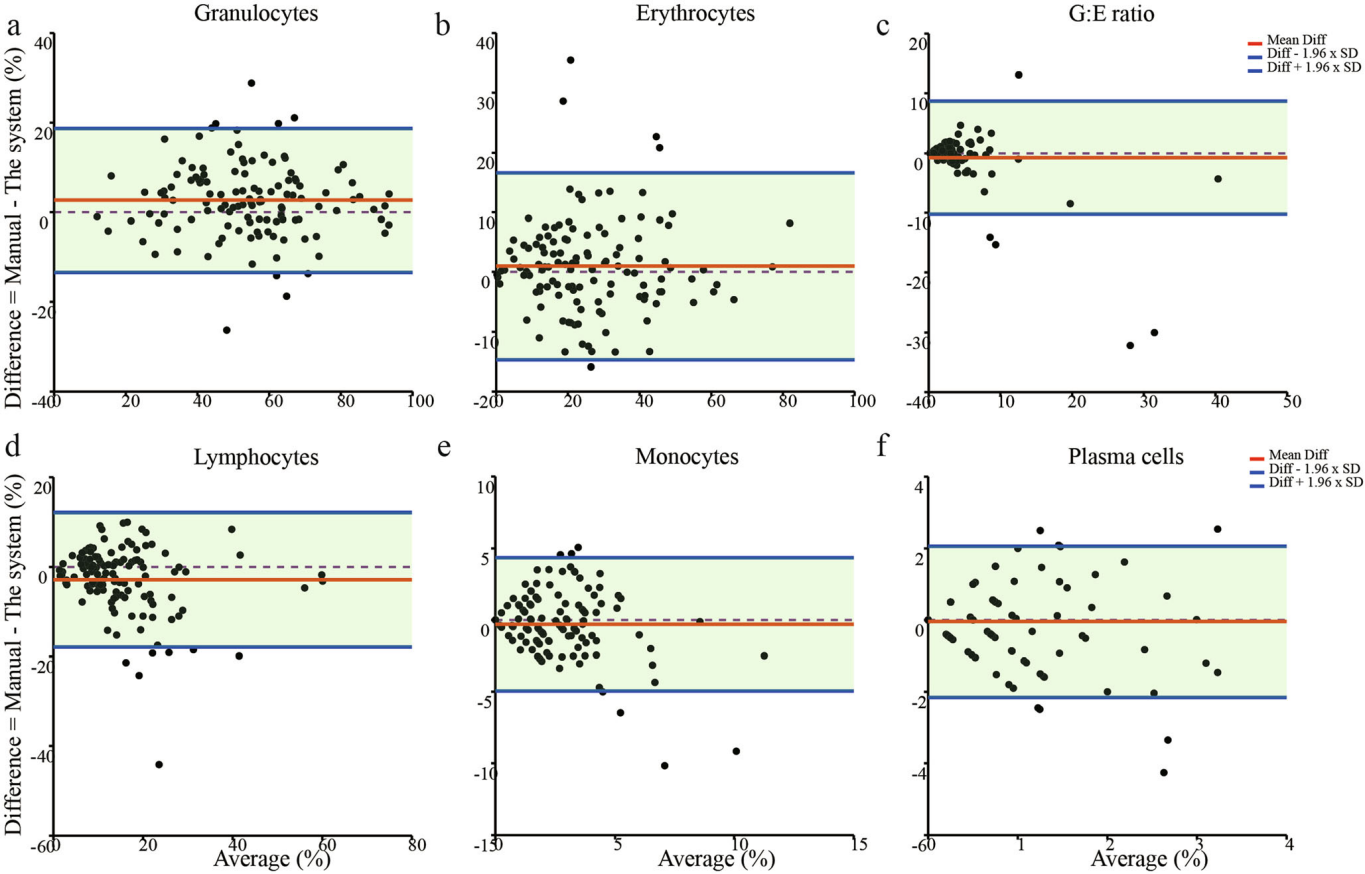
**Bland-Altman plots analysis of cell series proportions reviewed by the pathologists with the system and with manual different count.** a–f: Bland-Altman plots of granulocytes, erythrocytes, granulocytes: erythrocytes (G:E) ratio, lymphocytes, monocytes, plasma cells

Bland-Altman plot analysis showed that the variation of cell series proportions by pathologists with the two analysis methods were acceptable in 95% confidence for granulocytes, erythrocytes, and G:E ratio. Limit of agreement of granulocytes (%) = 2.676 ± 1.96 × 8.213, limit of agreement of erythrocytes (%) = 0.967 ± 1.96 × 7.981, limit of agreement of G:E ratio = −0.761 ± 1.96 × 4.861 (Fig. 6a–c). It is difficult to give the acceptable threshold range of lymphocytes, monocytes and plasma cells because of small cell proportion (Fig. 6d–f).

## Discussion

In this study, we developed an automatic system which was proved to be effective in performing complex morphology-based cell classification on high resolution digital images of BM smears.

The critical step of digital morphology analysis in hematology was digitization of smears. The resolution of the digital images of BM smear scanned by our system was validated for clinical application. Digital morphology analysis should use high optical magnification, if possible 100 ×. [8, 14] DM9600 is equipped with 10×, 40× and 100 × (oil) objectives, and it captures 100× images of WBCs and 50× images of RBCs with a reducing lens. Vision Hema is equipped with 10×, 50× (oil) and 100× (oil) objectives, and it also captures 100× images of WBCs and 50× images of RBCs and platelets (PLTs). [8] However, these systems were only used for peripheral blood smears. So far, no successful instrument is proven to be efficient for BM smear scanning. Although some studies showed that Zeiss Axio Imager Z2 [12] and Aperio AT2 scanner [9]) could be used for BM smear scanning and provide high-resolution images, they only used 40× objectives for their maximum magnification, which could not meet the needs of the morphology examination in the clinical applications, since the morphology examination required 100× views for observing detailed information inside the cells. Several systems for BM smear scanning are currently under investigation, such as Vision Bone Marrow (with 10×, 50× oil, 100× oil) [22] and Scopio Full Field BMA. [23] Scopio Full Field BMA uses oil-free lens and special illumination to produce low-magnification images and then reconstruct them into super-resolution images that of 100 x equivalent magnification, but in optical imaging, the numerical aperture of the objective lens limits the optical resolution of the microscope. More detailed cellular information cannot be obtained by digitally enlarging the images or increasing the magnification, for example, neutrophil cellular granules (0.2 μm) of WBCs could only be detected by high-quality oil-immersion lens. [14] Experienced pathologists will perceive subtle changes from cellular granularity and finely chromatin texture under the optical microscope. Our goal is to make cellular imaging infinitely closer to that of the optical microscope, and to maximize the detailed information inside cells, we are developing techniques for 3D cellular imaging. In addition, the technique of Scopio involves a large amount of computation and it affects the scanning and processing speed (<5 min/cm^2^). Though observation and classification of individual cells require high magnification, the WSI can be produced at relatively lower magnification. [14] The scanning time of our system for a BM smear which counting 500 cells was about 32 min/slide (with 40 × and 100 × oil), close to that of the DM9600 for peripheral blood slides (1.5 slides/h for 10 × 10 mm in 10× + 50×). [30] As far as we know, this is the first automated system successfully developed for digital scanning of BM smears.

The results of our initial validation data are convincing that the algorithm of the system can automatically identify common BM cells, the accuracy of cell classification was reaching 90%. Therefore, our system has the capacity to reduce working load of the manual BM smear examinations and improve the efficiency of morphology analysis. In addition, it also provides possibility of standardization across the lab and as a platform for real time telepathology consultation.

For cell classification, Choi et al. [10] developed a classifier and achieved an accuracy of 0.971 for ten classes with 2174 nonneoplastic cells, and Liu et al. [13] developed a classifier and reported an average recognition rate of 87.49% for five type cells with 8004 images. Despite the high accuracy of the classifier reported, they studied only a few types of cells which were easy to be distinguished, and the sample size tested was relatively small. Krappe et al. [12] developed a classifier for 16 different types of BM cells, and reported an overall accuracy of 66.3% with 46,189 cells, and the highest accuracy for cell types range from 76% to 94%. Chandradevan et al. [9] developed a system for 12 different types with 10,000 annotated cells from neoplastic and nonneoplastic cases, and reported a median total AUC of 0.98 ± 0.03 with nonneoplastic samples, and the median AUC for each class ranged from 0.960 (monocyte) to 1.00 (basophil). The results of the study further verified and expanded on the work of previous publications.

In terms of analysis ability of smears, the reliability coefficient of two different analysis methods were high for granulocytes, erythrocytes, and the results of cell series proportions by the two analysis methods were consistent for granulocytes, erythrocytes, and G:E ratio. Though the system has the capacity to analyze marrow smears and generate reports automatically, the results should be reviewed by the pathologists before it is final released. The diagnosis of BM smear should be made in the context of correlation with clinical information and relevant laboratory findings including immunophenotyping flow cytometry, molecular studies by FISH, cytogenetics or NGS analysis.

Several limitations should be addressed. In cell classification, challenge still remains in order to distinguish the immature cells among myeloblasts, promyelocytes due to the overlapping features of the spectrum of those cells. Cell number in different types of nucleated cells varied greatly, hence some cells were rarely found while some were abundant in BM smears. The morphological differences between types of cells can be subtle and some cell types were extremely rarely found in normal BM cell repertoire. The unbalanced cell numbers and lack of obvious morphological characteristics directly influenced the system’s ability to classify nucleated cells. Good sensitivities were obtained for cell types in which large sample sizes were collected and the morphological characteristics were clear and relatively more distinguishable, and poor sensitivities were obtained for cell types that were usually rarely found and collected. For instance, myeloblasts, promyelocytes and myelocytes are myeloid cells in different developing states, however there are no clear cutoffs between each type of them. In addition, the number of myeloblasts present in the collected smears were relatively low, and the criteria for identifying promyelocytes varied among different pathologists. Moreover, these three types of myeloid cells were difficult to distinguish from polychromatic erythroblast, lymphocyte, immature monocyte, lymphoblast/precursor B-cells during auto-classification. For eosinophils, basophils, plasma cell and others, due to their low ratio of nucleated cells in BM smears, the number of cells collected were not enough to accurately train the system. As erythroblasts, polychromatic erythroblast and orthochromatic erythroblast are morphologically similar to lymphocyte, proplasmocyte and plasma cell, when auto-classifying these types of cells, their similar morphological characteristics influenced the system’s ability to distinguish them and resulted in low sensitivities and high false negative rates. Matek et al. reported an algorithm achieved human-level recognition of blast cells in peripheral blood smears (precision of myeloblast 0.94). [31] Merino et al. reported qualitative morphological issues in lymphoid and blast cells and quantitative morphological features based on image analysis (geometric, color, and texture) may help to optimize morphology analysis. [32] While deep learning networks automatically extract cellular features without human intervention, next we will use these cellular features and research results to build new deep learning neural network algorithms, such as unsupervised learning, small sample learning, and the latest brain-like computational neural networks. The algorithm performance will continue to improve while accumulating more samples of BM smears. In addition, automatic appropriate field selection, stain quality, unbalanced cell number of smears would impact the system’s analysis ability of BM smear.

In summary, we developed and applied an automated system to perform differential cell count in BM smears in this pilot study. The system was validated effective in cell classification of BM smears. It has a potential capability in assisting examination of BM smear in clinical application.

## Electronic supplementary material

ESM 1 (PDF 1040 kb)

ESM 2 (XLSX 16 kb)

## Data Availability

Data needed are present in the supplement, other raw data generated by the system, if necessary, are available from the corresponding author. Xinyan Fu, Haiyan Gao and Jun Zhang had access to data sets of bone marrow smears and responsible for publication.
