# Supplementary material for: Developing and Preliminary Validating an Automatic Cell Classification System for Bone Marrow Smears: a Pilot Study
Source: J Med Syst. 2020 Sep 7;44(10):184. doi: 10.1007/s10916-020-01654-y (PMC7476995; doi:10.1007/s10916-020-01654-y)
Supplement: Supplementary file 1 — (PDF 1040 kb) [file 10916_2020_1654_MOESM1_ESM.pdf]

Electronic supplementary material

Article title: Developing and preliminary validating an automatic cell classification system for bone marrow smears: a pilot study

Journal name: Journal of Medicinal Systems

Author names: Hong Jin, B.S.Med; Xinyan Fu, MS; Xinyi Cao B.S.Hons; Mingxia Sun, B.S.; Xiaofen Wang, M.M.; Yuhong Zhong, M.M.; Suwen Yang, M.M.; Chao Qi, M.M.; Bo Peng B.S.Med; Xin He, M.M.; Fei He, M.M.; Yongfang Jiang, M.M.; Haiyan Gao, M.M.; Shun Li, M.E.; Zhen huang, M.S.; Qiang Li, M.E.; Fengqi Fang, PhD and Jun Zhang, PhD

Affiliation and e-mail address of the corresponding author:

Xinyan Fu, MS

Division of Medical Technology Development, Hangzhou Zhiwei Information and Technology Ltd. Hangzhou, 311121, China.

Email: 21018259@zju.edu.cn

or

Jun Zhang, PhD

Clinical laboratory, Sir Run Run Shaw Hospital, Zhejiang University School of medicine, Hangzhou, 310016, China.

Email: jameszhang2000@zju.edu.cn

or

Haiyan Gao, M.M.

Department of Hematology, The Second Hospital affiliated of Harbin Medical University, Harbin, 150086, China.
Email: 21448840@qq.com

Table of Contents:

Online Resource 1. Demo video of the system

Online Resource 2. Detail description of workflow of the system

Online Resource 3. Table 1 Information of smears

Online Resource 1. Demo video of the system

https://www.morphogo.com/en/product-details.html

Online Resource 2. Detail description of workflow of the system

Preparation

To prepare for image acquisition with the acquisition terminal, users need to enter in patient information, such as smear number, medical record number, name, age, gender and date. Print a QR code with smear information and stick it on the BM smear (see **Figure 1a**). Then, inserting the smear into a global view box to set analysis number of nucleated cells and select analysis area of interest for scanning (see **Figure 1a**). The selection of analysis area was critical since it would directly impact the quality of cell images captured and it required technical skills and experiences. The system supports to select an appropriate analysis area (40× and 100×) automatically, or randomly select an appropriate analysis area (40×) and three regions of interest (100×) within the appropriate analysis areas on the slide manually by the user (see **Figure 7，8**). Areas around the particles and the feathered edge of the film must be examined carefully (see **Figure 9, 10**). Besides the area mentioned above, the head, the tail and sides of the smear should also be scanned for existences of abnormal cells, clumped immature cells, and cancer cells including myeloma, lymphoma and metastasis (see **Figure 9***)*. The number of cell count for a smear was set to 500 generally, with the exception of very low or very high degree of myelodysplasia. For smear with myelodysplastic, it could count to 1000 nucleated cells for more detailed and accurate clinical information. The system supports counting of 5000 cells or more (see **Figure 3，10**).

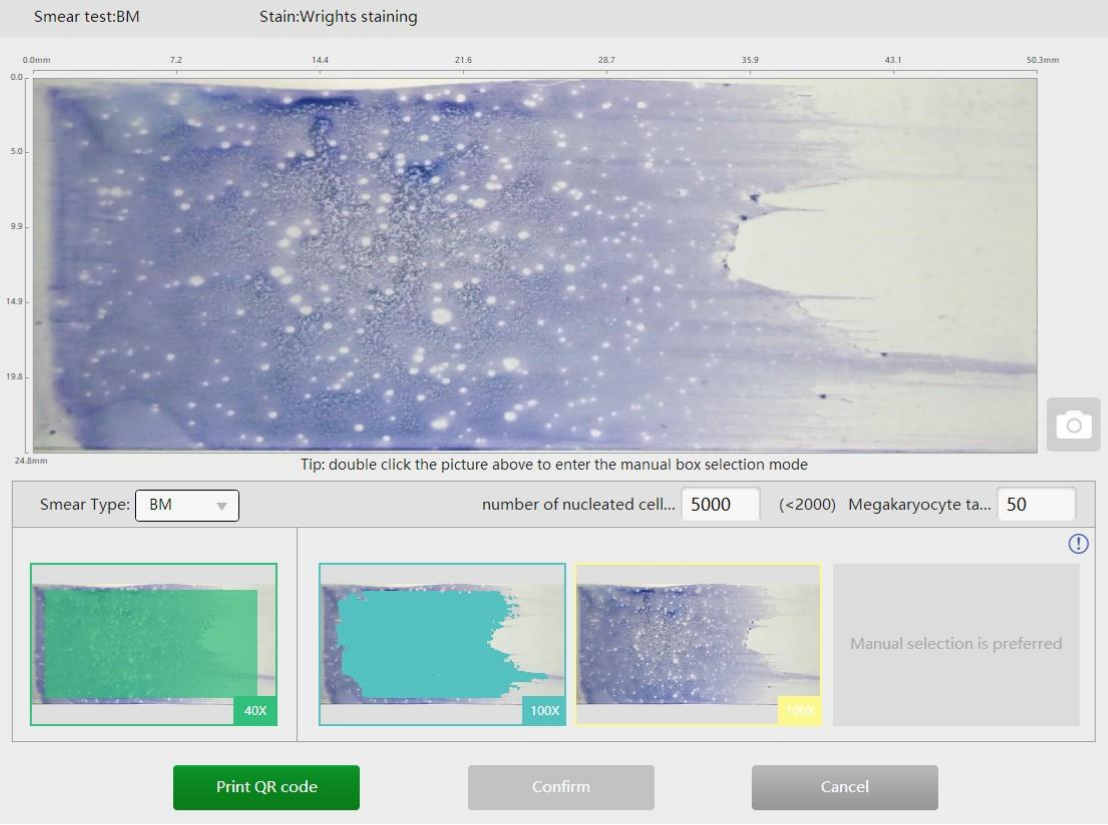

Figure 7 Automatic appropriate analysis selection（40× plus 100×）of a smear with lymphoma. Automatic area selection algorithm imitates a pathologist's behavior to choose an area where nucleated cells are likely to be non-overlapping and distributed evenly. The area in green is an appropriate analysis area (40×) automatically selected by the algorithm，and the area in cyan is regions of interest (100×) automatically selected by the algorithm.

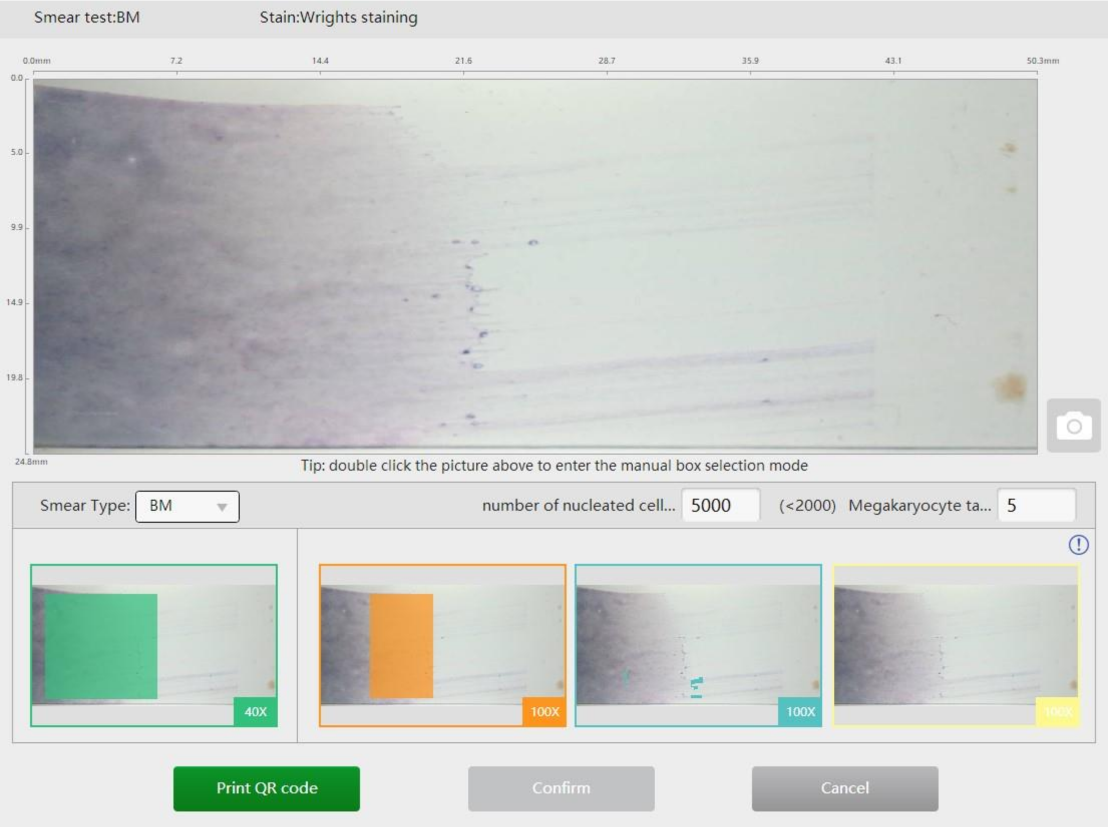

Figure 8 Manual appropriate analysis selection （40× plus 100×） of a smear with multiple myeloma. The area in green is appropriate analysis area (40×) manually selected by a pathologist, and the area in yellow is regions of interest (100×) manually selected by a pathologist. The number of regions of interest (100×) can be set manually is up to three.

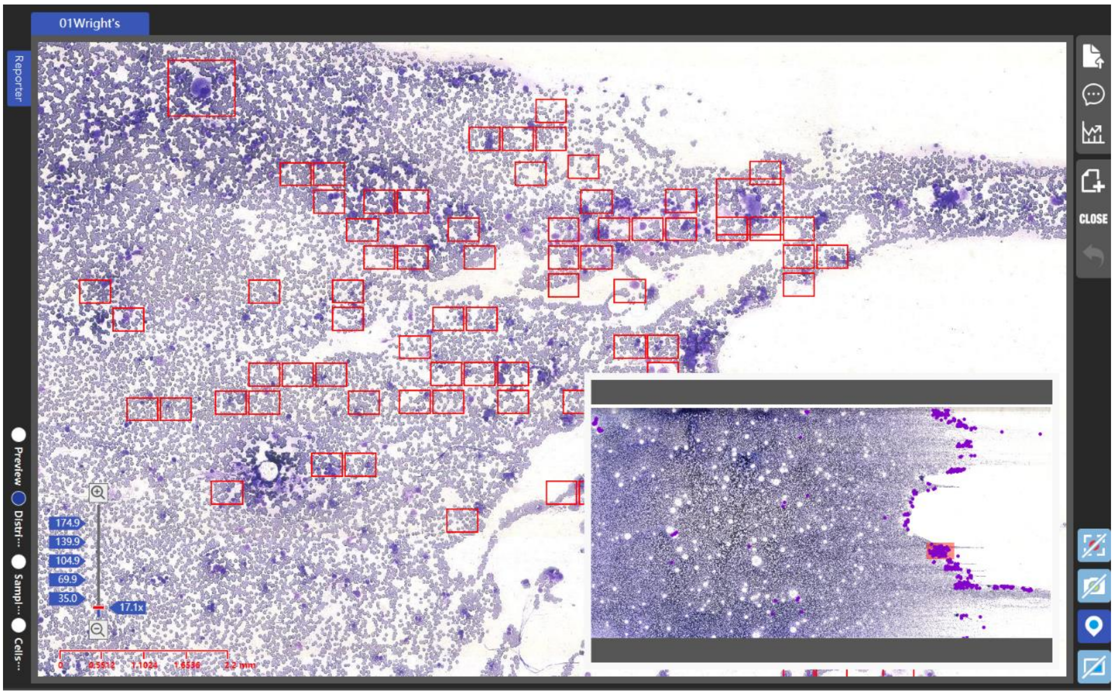

Figure 9 Automatic appropriate analysis selection （40× plus 100×） of a smear with lymphoma (count 558 cells). Red-colored boxes on the digital slide (40×) are the image fields actually captured by 100× objective lens. These fields are near the fat drops or close to the tail of the slide.

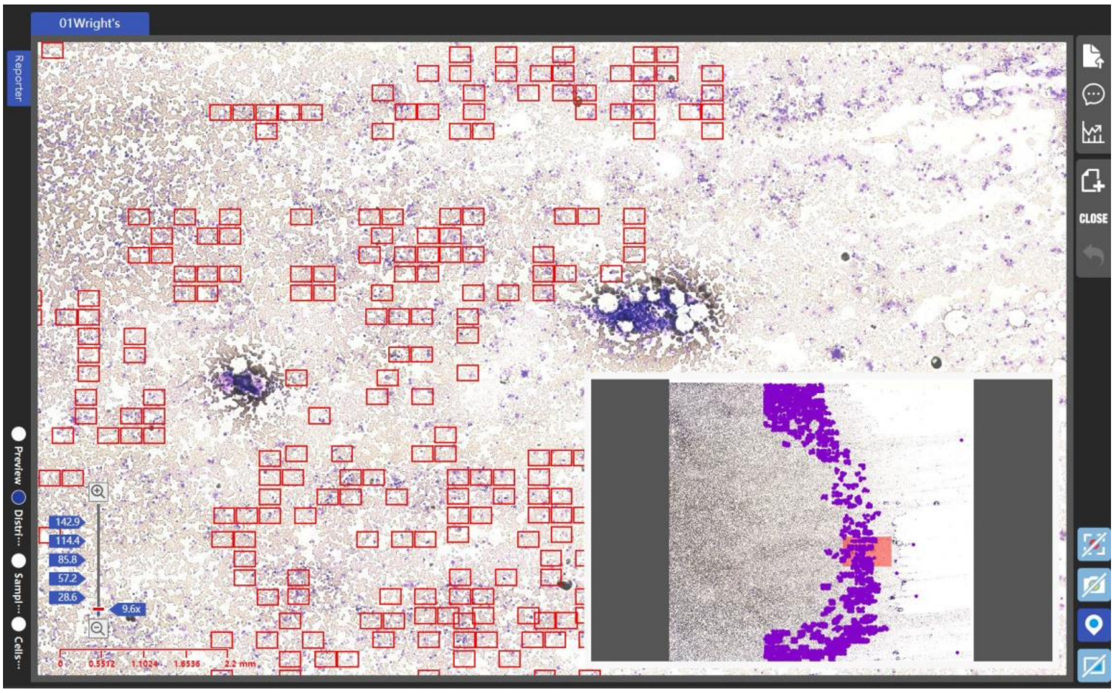

Figure 10 Manual appropriate analysis selection （40× plus 100×） of a smear with multiple myeloma (count 4980 cells). Though appropriate analysis area (40×) and regions of interest (100×) were selected manually, the algorithm automatically finds appropriate area for a 100× field on the slide. Red-colored boxes on the digital slide (40×) are the image fields actually captured by 100× objective lens. These fields are near the fat drops or close to the tail of the slide.

Acquisition of digital whole slide imaging (40×)

Before WSI scanning, the system takes 24 focusing references points (4×6, interval space 6 mm) on the analysis area of the smear to calculate focal plane of the smear. Then, the software performs a precise focusing process on the first cell located in the image with the autofocusing algorithm, and starts a scanning across the analysis area of the smear. Finally, all the images captured were assembled into a seamless WSI by the software.

Acquisition of high magnification cell images (100×)

When the WSI scanning was completed, the system lens switched from 40 × to 100 × oil-immersed objective automatically. The drops of oil on the slide is strictly controlled and monitored by an oil dropping sensor. Then, high magnification image of cells is acquired automatically. When the number of nucleated cells in the images meets the requested number of pre-determined, the process of image acquisition and cell classification by the system is done and stopped automatically.

Review result of cell count and issue a report

The results of cell classification could modify by the user in the acquisition terminal. Or upload the cell images and analysis results to the review terminal for experienced users to review and issue a BM report.
